# Infective endocarditis caused by *Cardiobacterium valvarum*

**DOI:** 10.1099/acmi.0.000040

**Published:** 2019-06-26

**Authors:** Yohei Washio, Shun-ichiro Sakamoto, Ryoichi Saito, Takahito Nei, Masayo Morishima, Akihiro Shinoyama, Ayaka Tashiro, Ryoji Sugimoto

**Affiliations:** ^1^ Nippon Medical School Hospital, Department of Clinical Laboratory, Bunkyo, Tokyo, Japan; ^2^ Nippon Medical School Hospital, Department of Cardiovascular Surgery, Bunkyo, Tokyo, Japan; ^3^ Department of Molecular Microbiology, Graduate School of Medical and Dental Sciences, Tokyo Medical and Dental University, Bunkyo, Tokyo, Japan; ^4^ Nippon Medical School Hospital, Department of Infection Control and Prevention, Bunkyo, Tokyo, Japan

**Keywords:** infectious ecdocarditis, *Cardiobacterium valvarum*, genotyping, HACKE group

## Abstract

We report a case with infective endocarditis (IE) due to *Cardiobacterium valvarum *. The patient was a 57-year-old male, who was referred to our hospital based on suspected IE detected by transthoracic echocardiography at a neighbourhood clinic. Three sets of blood cultures obtained on admission yielded positive results, and revealed rather slender and linear Gram-negative bacilli with a rosette formation that dyed minimally, with a pale white appearance. Although no isolates were identified by conventional methods, *C. valvarum* was ultimately identified by 16 S ribosomal RNA genotyping. HACEK group strains are difficult to identify by conventional methods. Therefore, if Gram-negative bacilli are isolated from IE patients, 16 S ribosomal RNA genotyping will be necessary. Furthermore, IE due to *C. valvarum* is very rare. We thus discuss our case in comparison with previous reports.

## Introduction

*Cardiobacterium* spp. are classified into the HACEK group, resident oral Gram-negative bacilli that are widely known to be causative pathogens of infective endocarditis (IE) [1–3]. These organisms include *Haemophilus paraphrophilus *, *Aggregatibacter actinomycetemcomitans *, *Cardiobacterium hominis *, *Eikenella corrodens* and *Kingella kingae* [1, 3]. The organisms have been estimated to have caused infection in <5 % of IE patients according to previous reports [2, 4]. There are still many unclear points in the clinical description and full portrait of HACKEK IE because of its relative rarity and because there are few large case series and case reports [1, 5].

The genus *Cardiobacterium* was established as a member of this category in 1964 but included only one species, *Cardiobacterium hominis* [6]. However, in 2004 *Cardiobacterium valvarum* was then reported to have been isolated by culture from a patient with IE [7], and its biochemical properties were determined. Only 16 reports of infective endocarditis due to *C. valvarum* can be found when searching online (PubMed, 12 June 2019). Herein, we present this case of IE caused by *C. valvarum *.

### Case Presentation

**Fig. 1.**
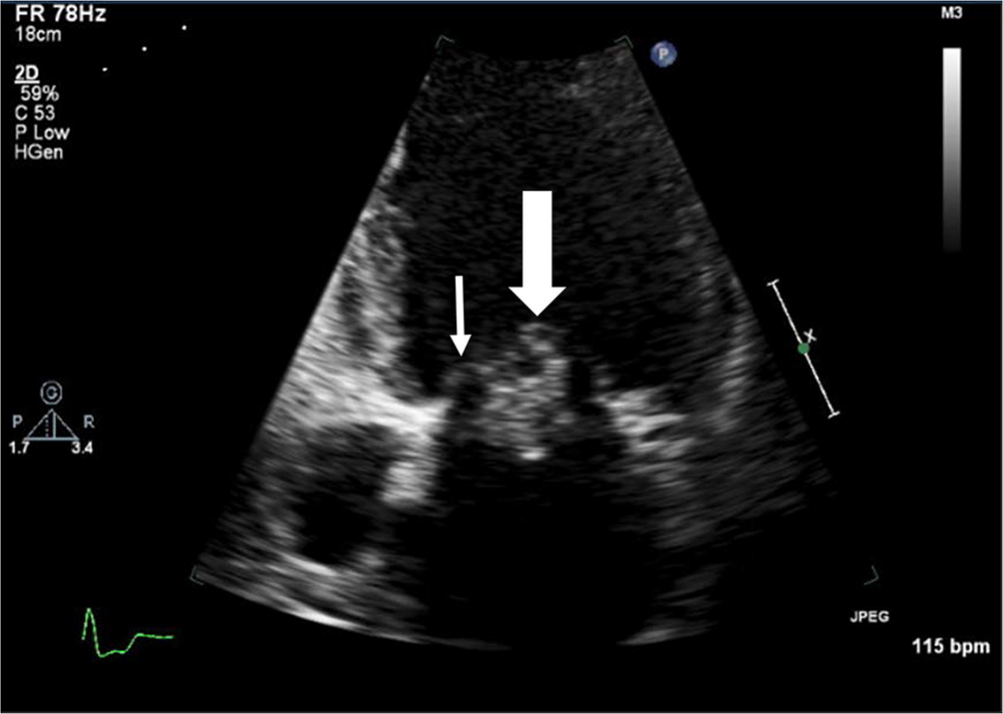
Echocardiogram performed on the first day in hospital. A movable mass (large white arrow, 23.7mm×14.9mm) can be seen on the anterior mitral leaflet (small white arrow), and this caused the valve annulus to adhere to the valve leaflet.

A 57-year-old apparently healthy Japanese male, followed by a neighbourhood physician for hypertension, underwent a regular blood examination that revealed an elevated white blood cell count (12 600 μl^−1^) and elevated C-reactive protein (CRP; 2.97 mg dl^−1^). Both persisted for the next month. He was referred to a cardiologist at our hospital and received further examinations. The laboratory data are shown in Table 1. Subsequently, an echocardiogram revealed a wart on the anterior mitral valve with regurgitation (Fig. 1). There were no symptoms other than breathlessness on exertion and there were no embolic symptoms such as Osler’s nodes. He was referred to the department of cardiac surgery at our hospital for suspected IE and was admitted directly because a transesophageal cardiac echocardiogram showed mitral valve destruction with a wart (Fig. 1).

**Table 1.** Laboratory data on admission

Blood cell counts	
White blood cells	12 600 μl^−1^
Neutrophils	77.1%
Lymphocytes	17.0%
Eosinophils	1.0%
Red blood cells	447×104 µl^−1^
Haemoglobin	11.9 g dl^−1^
Platelets	26.1×104 μl^−1^
Biochemical parameters	
Aspartate aminotransaminase	19 IU l^−1^
Alanine aminotransferase	21 IU l^−1^
Lactose dehydrogenase	272 IU l^−1^
γ-glutamyl transferase	31 IU l^−1^
Sodium	138 meq l^−1^
Potassium	4.7 meq l^−1^
Chloride	108 meq l^−1^
Blood urea nitrogen	17.3 mg dl^−1^
Creatinine	1.18 mg dl^−1^
Total protein	6.3 g dl^−1^
Albumin	3.1 g dl^−1^
Plasma glucose	116 mg dl^−1^
Haemoglobin A1c	6.2%
Serum inflammatory marker	
C-reactive protein	2.97 mg dl^−1^

## Results

Emergency surgery was performed on the day of admission, and blood samples for microbiological culture were obtained. Subsequently, we isolated unidentified Gram-negative bacilli from whole-blood culture samples.

### Microbiological and molecular results

**Fig. 2.**
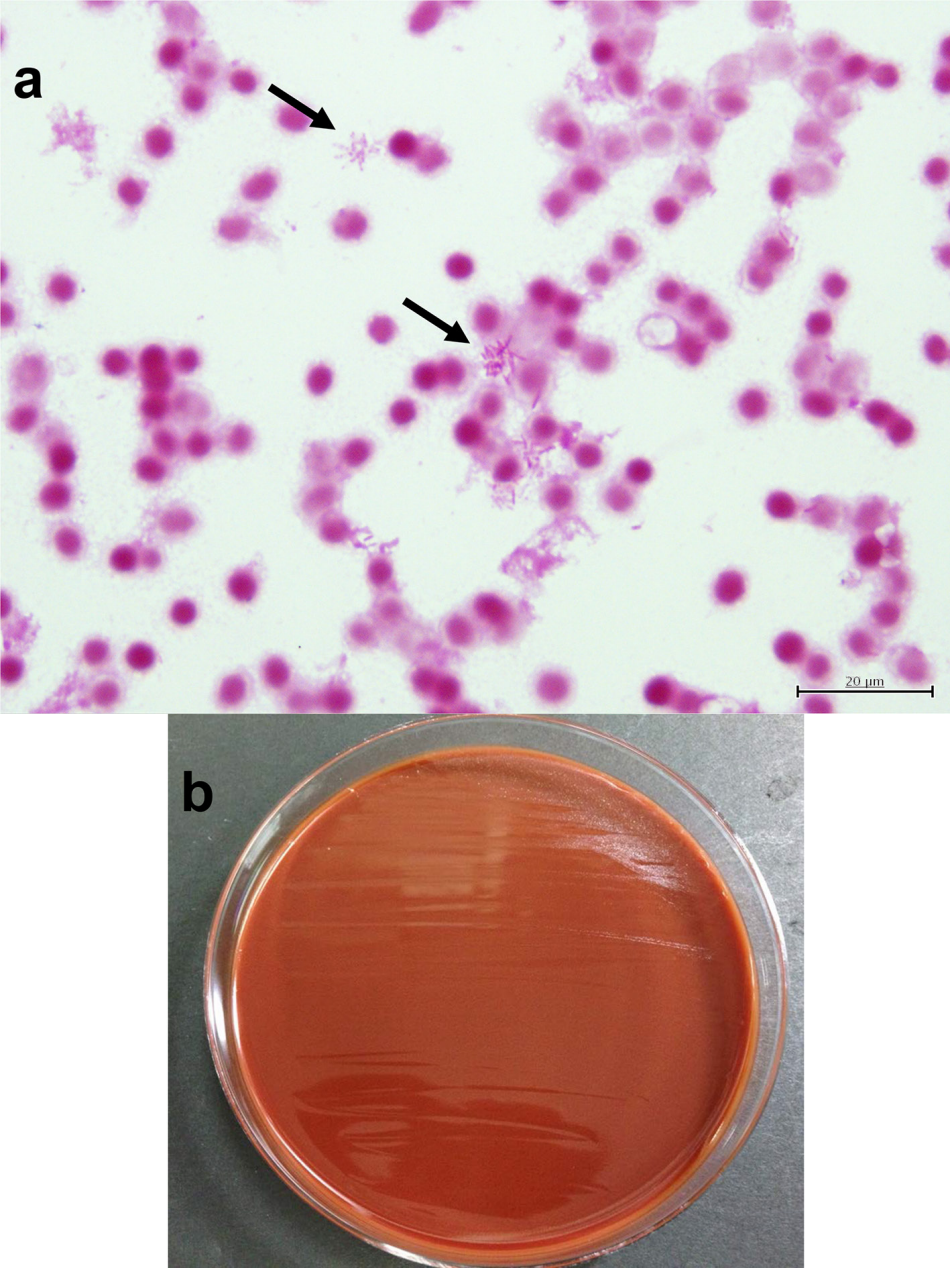
(a) Smear photograph of bacilli isolated from a blood culture sample obtained on the day of admission. We identified the causative organism based solely on an aerobic culture bottle that yielded a positive result on the fifth day in hospital. We also performed direct staining of the culture bottle contents using Bartholomew and Mittwer’s method, which revealed slightly elongated and mildly stained Gram-negative bacilli with a rosette formation (black arrows). (b) Isolation of colonies from the blood culture sample. The colonies grew slowly, but we were able to isolate a few tiny white colonies, with a diameter of 0.1 mm, after 72 h of capneic culture.

We used BACTEC Plus Aerobic/F culture vials and anaerobic/F culture vials (Becton Dickinson, Tokyo, Japan) for blood culture sampling and incubation (BACTEC FX, Becton Dickinson) at 35 °C. The blood culture sample obtained before antibiotic administration only yielded a positive result by aerobic culture on the fifth day in hospital. Direct staining from the culture bottle using Bartholomew and Mittwer’s method showed slightly elongated and lightly stained Gram-negative bacilli with a rosette formation (Fig. 2a). For identification, we used 5 % sheep serum agar (Eiken, Tokyo, Japan), MacConkey agar (Oriental Yeast Co. Ltd, Tokyo, Japan) at 37 °C under aerobic incubation conditions. Furthermore, assuming the presence of *Haemophillus* spp., we also used chocolate agar (Becton Dickinson, Tokyo, Japan) at 37 °C under 5 % carbon dioxide incubation conditions.

Neither aerobic nor anaerobic growth was observed with incubation, although we isolated tiny white colonies with diameters of 0.1 mm at 72 h with capneic culture (Fig. 2b). Firstly, we identified these colonies as *Pasteurella multocida* using a commercial identification kit (BD BBL CRYSTAL N/H, Becton Dickinson, Tokyo, Japan). However, this identification was not consistent with the lack of growth on blood agar. Ultimately, we accurately identified the strain with 16S ribosomal RNA genotyping using universal primers 8 UA (5′-AGAGTTTGATCMTGGCTCAG-3′) and 1485B (5′-ACGGGCGGTGTGTRC-3′), as previously described [8], and a similarity search was conducted using the blast program (DDBJ, Shizuoka, Japan) and Ezbiocloud (https://www.ezbiocloud.net/). The 16S rRNA gene sequence obtained (GenBank accession no. LC383426; 1265 bp) showed highest similarity (with 99.68 %; 1261/1265 bp) to that of the *C. valvarum* type strain (GenBank accession no. AF506987) (Table 2). The results (1265 bp) showed 99.6 % (1261/1266) similarity to the type strain (GenBank accession no. AF506987) of *C. valvarum *. Determination of minimum inhibitory concentrations (MICs) was performed by microbroth dilution antimicrobial susceptibility testing using commercial plates (MicroScan Walkaway 96 Plus, Siemens, Inc. Berlin, Germany), although we were not able to detect the MICs due to the growth defect.

**Table 2.** Summary of identification conducted by Ezbiocloud using 16S rRNA gene sequences

Rank	Name	Strain	Accession no.	Similarity (%)	Mismatch/total nt	Completeness (%)
1	* Cardiobacterium valvarum *	MDA3079	AF506987	99.68	4/1265	99
2	* Cardiobacterium hominis *	ATCC 15826	ACKY01000036	96.36	46/1265	100
3	* Suttonella ornithocola *	NCTC 13337	UHIC01000001	93.12	87/1265	100
4	* Suttonella indologenes *	ATCC 25869	M35015	92.26	97/1253	99.9
5	* Dichelobacter nodosus *	VCS1703A	CP000513	90.5	120/1263	100

These results indicate bacterial species with similarity of >90% compared to the 16S rRNA gene sequence generated by the case (GenBank accession no. LC383426; 1265 bp).

## Discussion

Han *et al*. first reported an IE case due to *C. valvarum* in 2004 [7]. *C. valvarum* is a rare bacterium that has only been reported in 11 cases to our knowledge [7, 9–18]. These 11 cases were all identified by 16S ribosomal RNA genotyping. In these case reports, this organism caused infectious diseases more frequently in males than in females (11 versus 2, respectively), and the patients’ ages ranged from 28 to 72 years. With regard to cardiac valve damage, there were nine cases with aortic valve damage, two with mitral valve damage, one with pulmonary vein valve and one with tricuspid valve damage. The onsets in these cases were not only closely related to dental treatment histories, but also to cardiac malformations and past medical history of cardiac surgical intervention. Moreover, most *C. valvarum* cases tended to be afebrile and free of physical symptoms.

From the microbiological view point, *C. valvarum* has oxidase but not catalase activity. The glycolytic actions of *C. valvarum* were exerted on fructose, mannose and sorbitol. These actions are the same as those of *C. hominis* [19]. Han *et al*. reported that *C. valvarum* can be distinguished from *C. hominis* by its capacity to glycosylate maltose, mannitol and sucrose [7]. However, descriptions of these properties vary markedly among previous reports, and no consensus regarding glycolytic actions has yet been reached [12, 15, 16]. We consider the biochemical properties to lack confirmation because very few infections caused by this organism have been reported. We hope to establish features that would allow *C. valvarum* to be distinguished from *C. hominis *, because precise identification can only be achieved correctly by base sequencing. On the other hand, the growth of *C. valvarum* is thought to be slower than that of *C. hominis* [19], and growth characteristics are generally considered to be reliable with the use of blood agar or chocolate agar, although growth patterns vary among agar manufacturers.

Identification now depends on solely on gene sequencing for 16S ribosomal RNA. Various methods of identifying *C. valvarum* have been attempted, but using commercial kits carries a high risk of misidentification. *P. multocida* and *Haemophilus parainfluenzae ,* in particular, have often been falsely identified by commercial kits [7, 12, 13, 15, 16]. In our case, before 16S rRNA sequencing, we attempted identification using a commercial kit (BD BBL CRYSTAL N/H, Becton Dickinson, Tokyo, Japan), but the result for this indicated that the sample was *P. multocida *. A previous report showed that our detection kit indicated *Cardiobacterium* spp. [7], but the details were unclear and the possibility of precise identification remain uncertain. On the other hand, mass spectrometry may be feasible for identifying this organism. Previous reports have shown that mass spectrometry can not only detect the genus, but also the species in colonies growing on agar, but the homologous score was incomplete [17].

*C. valvarum* reportedly shows sufficient susceptibility to various antibacterial agents [7, 10, 12, 13, 15, 18]. However, there are reports of strains showing resistance to sulfamethoxazole/trimethoprim [16], and it is necessary to accumulate future data on drug susceptibilities. Although we could not conduct drug susceptibility tests for various antimicrobials, the patient’s improvement with the administration of ceftriaxone is a testament to *C. valvarum* showing little, if any, drug resistance.

Because *C. valvarum* can only be identified by determining the base sequence, the problem of not being able to identify this organism accurately was also raised in a previous report. Hagiya *et al*. suggested including *C. valvarum* among bacterial IE organisms, such as *C. hominis* [9]. Therefore, further accumulation of data is needed to improve the accuracy of identification.

### Conclusion

We experienced a case with IE due to *C. valvarum *, which is a relatively rare organism. This patient had no other cardiac malformations and did not have a history of previous dental treatment. At present, it is difficult to identify *C. valvarum* with a conventional test kit. Base sequencing is necessary, but gathering patient information is also necessary to fully understand this organism.
